# Coenzyme A, protein CoAlation and redox regulation in mammalian cells

**DOI:** 10.1042/BST20170506

**Published:** 2018-05-25

**Authors:** Ivan Gout

**Affiliations:** Institute of Structural and Molecular Biology, University College London, Gower Street, London WC1E 6BT, U.K.

**Keywords:** coenzyme A, metabolic control analysis, oxidation–reduction, post-translational modification

## Abstract

In a diverse family of cellular cofactors, coenzyme A (CoA) has a unique design to function in various biochemical processes. The presence of a highly reactive thiol group and a nucleotide moiety offers a diversity of chemical reactions and regulatory interactions. CoA employs them to activate carbonyl-containing molecules and to produce various thioester derivatives (e.g. acetyl CoA, malonyl CoA and 3-hydroxy-3-methylglutaryl CoA), which have well-established roles in cellular metabolism, production of neurotransmitters and the regulation of gene expression. A novel unconventional function of CoA in redox regulation, involving covalent attachment of this coenzyme to cellular proteins in response to oxidative and metabolic stress, has been recently discovered and termed protein CoAlation (S-thiolation by CoA or CoAthiolation). A diverse range of proteins was found to be CoAlated in mammalian cells and tissues under various experimental conditions. Protein CoAlation alters the molecular mass, charge and activity of modified proteins, and prevents them from irreversible sulfhydryl overoxidation. This review highlights the role of a key metabolic integrator CoA in redox regulation in mammalian cells and provides a perspective of the current status and future directions of the emerging field of protein CoAlation.

## Coenzyme A biosynthesis and degradation in eukaryotic cells

**Figure 1.**
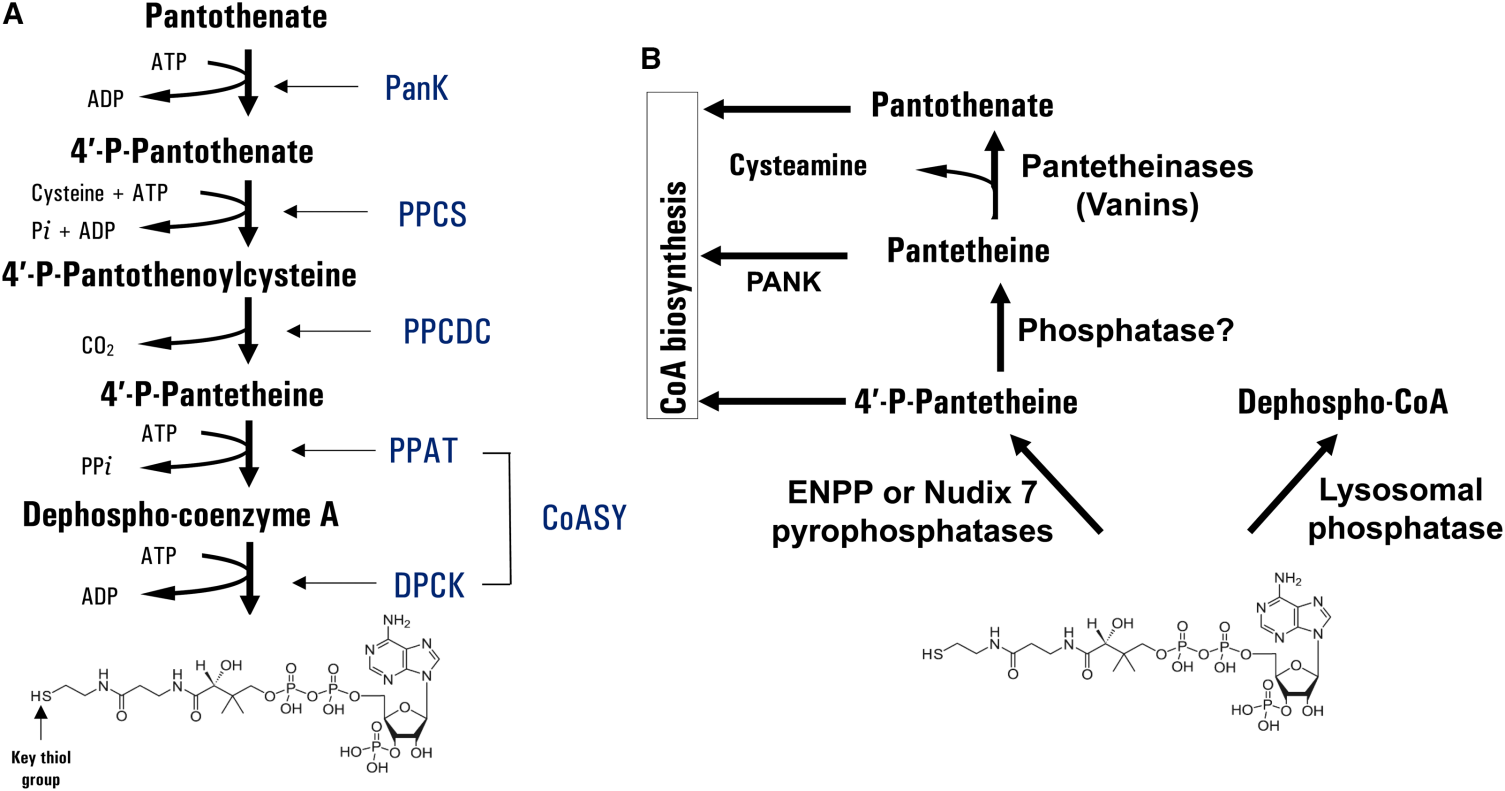
Biosynthesis and degradation of CoA in mammalian cells. (**A**) The conventional *de novo* and alternative pathways of CoA biosynthesis are shown. (**B**) CoA degradation involves phosphodiesterases, phosphatases and pantetheinases.

Coenzyme A (CoA) is a fundamental cofactor in all living organisms. It has a unique chemical structure which allows the diversity in biochemical reaction products and regulatory mechanisms. A classical pathway for CoA biosynthesis involves five enzymatic steps that are highly conserved from prokaryotes to eukaryotes and utilise pantothenate (vitamin B5), adenosine triphosphate (ATP) and cysteine (Figure 1A) [1]. The pathway is initiated by pantothenate kinase (PANK), which converts pantothenate into 4′-phosphopantothenate. 4′-Phosphopantothenoylcysteine synthase (PPCS) and phosphopantothenoylcysteine decarboxylase (PPCDC) catalyse the formation of 4′-phosphopantothenoylcysteine and 4′-phosphopantetheine (4′-PP), respectively. The last two steps in the CoA biosynthetic pathway are catalysed by CoA synthase (CoASy), which possesses two enzymatic activities: 4′-PP adenyltransferase (PPAT) and dephospho-CoA kinase (DPCK). An alternative route for CoA biosynthesis has been recently uncovered under conditions when the conventional *de novo* pathway is impaired and the level of intracellular CoA is significantly reduced [2]. It has been proposed that intracellular CoA pools could be replenished through the degradation of external sources of CoA (diet or culture medium) by ectonucleotide pyrophosphatases (ENPPs) to 4′-phosphopantetheine (P-PanSH), which is then transported into a cell and incorporated in the CoA biosynthetic pathway downstream of PPCDC [3]. The proposed mechanism requires further validation, especially the existence of dedicated P-PantSH transporters on cell membranes.

The biosynthesis and homeostasis of CoA is controlled at different levels: transcription of genes encoding biosynthetic enzymes, regulation of enzymatic activities by a feedback mechanism, signalling pathways, degradation of CoA and interconversion between CoA and its thioester derivatives. Various extracellular stimuli, such as nutrients, hormones of metabolic homeostasis, intracellular metabolites and stress, were found to regulate the total level of CoA in mammalian cells. It is reduced in to insulin, glucose, pyruvate and fatty acids, whereas glucagon, glucocorticoids and oxidative stress have an opposite effect [4–8]. PANK is the master regulator of CoA biosynthesis. There are four PANK isoforms in mammals, which exhibit a different pattern of expression, subcellular localisation and mode of regulation, allowing them to sense and control levels of CoA/CoA derivatives in various cellular compartments [9]. The expression and activity of the PANK proteins are governed by multiple mechanisms. Feedback inhibition by CoA/CoA thioesters (primarily acetyl CoA) is the principal mechanism for controlling the activity of mammalian PANK.

Regulation of other enzymes in the CoA biosynthetic pathway, especially PPCS and PPCDC, is less understood. The PPAT and DPCK activities of CoA synthase were found to be strongly induced by phospholipids [10]. The identification of CoA synthase in signalling complexes with ribosomal S6 kinase (S6K), class 1A phosphatidylinositol 3-kinase (PI3K), Src family kinases and enhancer of mRNA-decapping protein 4 (EDC4) suggests the regulation of CoA biosynthesis via signal transduction pathways and stress response [11–14].

The total cellular CoA content is also controlled by degradation, involving phosphodiesterases, phosphatases and pantetheinases (Figure 1B) [15]. The degradation of CoA results in the generation of products which are known intermediates in the biosynthetic pathway. CoA was found to be dephosphorylated at the 3′ position of the ribose ring by a lysosomal alkaline phosphatase, leading to the formation of dephospho-CoA [16]. Several peroxisomal and mitochondrial nucleotide diphosphate hydrolases (Nudix) were shown to hydrolyse CoA and acyl CoA thioesters to yield 3′,5′-adenosine mononucleotide and 4′-phosphopantetheine or acyl-phosphopantetheine [17,18]. The degradation of extracellular CoA was found to be mediated by ENPP, which functions as a phosphodiesterase and produces 3′,5′-ADP and 4′-phosphopantetheine. Dephosphorylation of 4′-phosphopantetheine by phosphatases produces pantetheine, which is further degraded to pantothenate and cysteamine by pantetheinases [19] (Figure 1B). Produced pantothenate may re-enter the CoA biosynthetic pathway or be excreted.

## CoA content and subcellular localisation

The estimated CoA levels in mammalian cells and tissues span more than a 10-fold range. Liver, heart and brown adipose tissue have the highest CoA levels, followed by kidney and brain. The CoA pool is largely made up of CoASH, and acetyl CoA is the largest component of the acyl CoA pool. The subcellular distribution of CoA in mammalian cells reflects the variety of processes in which it is implicated. The concentration of CoA in mitochondria and peroxisomes are in the range of 2–5 and 0.7 mM, respectively, whereas levels of cytosolic and nuclear CoA are significantly lower, ranging from 0.05 to 0.14 mM [1]. CoA is a large and charged molecule, therefore, it must be distributed to subcellular organelles via dedicated transporters. High-affinity transporters for CoA and dephospho-CoA were identified on mitochondrial and peroxisomal membranes [20,21].

## Cellular functions of CoA and its thioester derivatives

**Figure 2.**
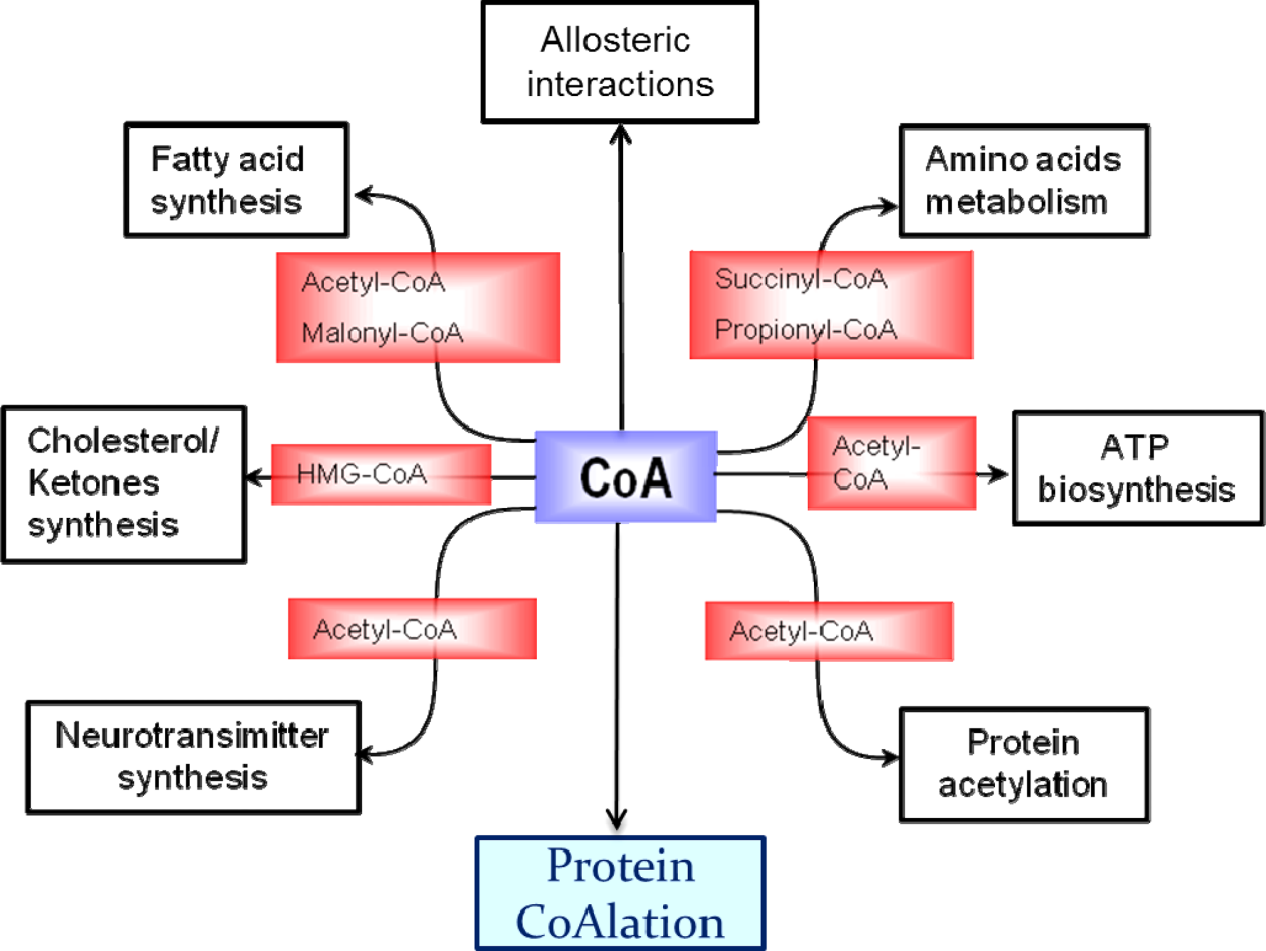
Cellular functions of CoA and its derivatives. CoA thioester derivatives are implicated in diverse cellular functions, including the Krebs cycle, ketogenesis, biosynthesis of cholesterol and acetylcholine, the degradation of amino acids, the synthesis and oxidation of fatty acids, biosynthesis of neurotransmitters and the regulation of gene expression. Protein CoAlation is a novel, unconventional function of CoA in redox regulation and antioxidant defence.

CoA and its thioester derivatives play important roles in numerous biosynthetic and degradative pathways of cellular metabolism, allosteric interactions and the regulation of gene expression. These include synthesis and oxidation of fatty acids, the Krebs cycle, ketogenesis, biosynthesis of cholesterol and acetylcholine, degradation of amino acids, regulation of gene expression and cellular metabolism via protein acetylation and others (Figure 2) [1,22,23]. Abnormal biosynthesis and homeostasis of CoA and its derivatives are associated with various human pathologies, including diabetes, Reye's syndrome, cancer, vitamin B12 deficiency and cardiac hypertrophy [24–26]. Genetic studies in human and animal models revealed the importance of the CoA biosynthetic pathway for the development and functioning of the nervous system [27,28]. Mutations in the human PANK2 and COASY genes were found to be associated with a degenerative brain disorder, termed neurodegeneration with brain iron accumulation (NBIA).

Although *de novo* CoA biosynthesis is an evolutionary conserved biochemical process, significant structural and regulatory differences between microbial and human biosynthetic enzymes make the CoA biosynthetic pathway an attractive target for the development of novel antibiotics.

## CoA: a major low-molecular-weight thiol in mammalian cells

The role of the CoA thiol group in the production and function of various thioester derivatives has been extensively studied since the discovery of this coenzyme in the middle of last century. In contrast, the contribution of the CoA thiol moiety towards redox regulation and antioxidant defence has yet to be established.

Mammalian cells contain high levels of low-molecular-weight (LMW) thiols that provide protection against a variety of reactive oxygen, nitrogen and electrophilic species (ROS, RNS and RES) generated inside cells by incomplete reduction of molecular oxygen, dysregulation of metabolic processes or produced during the detoxification of xenobiotic and endobiotic compounds. High levels of ROS, RNS and RES have the potential to damage cellular macromolecules, including proteins, nucleic acids, lipids and carbohydrates [29]. In contrast, low levels of reactive chemical species can act as second messengers and key regulators in signal transduction and metabolic pathways [30].

In mammalian cells and tissues, glutathione (GSH) is the most abundant LMW thiol with concentrations ranging from 0.5 to 10 mM [31]. GSH is also the most studied and best characterised thiol that functions to protect cellular macromolecules from oxidative damage and detoxify xenobiotics and toxic endogenous products, such as aldehydes, quinones, epoxides or alkyl hydroperoxides. Other biologically relevant LMW thiols include cysteine, homocysteine, taurine, lipoic acid and CoA [29]. The wide variety in structures of LMW thiols allows them to participate in diverse biochemical reactions, and it is not surprising that their cellular functions vary widely.

The redox functions of CoA in mammalian cells under physiological and pathophysiological conditions are not well understood. The relatively high p*K*_a_ of the CoA thiol (∼9.8) at physiological pH protects it from oxidation to the sulfenic acid state (CoASOH) [32]. To perform a nucleophilic attack, the p*K*_a_ of the CoA thiol needs to be decreased. The reactivity of CoA in cellular redox processes can be enhanced by complexing with the enzyme(s) which can reduce the p*K*_a_ value of its thiol and facilitate covalent modification of cellular targets by CoA, as reported for GSH in complex with glutathione transferases [33]. The enzyme(s) possessing this activity has yet to be identified.

The bulk of CoA in mammalian cells exists in reduced (CoASH) and thioster (acyl CoA) forms. CoA disulfides (CoASSCoA) or mixed disulfides with other LMW thiols (such as cysteine and GSH) have been identified in mammalian cells, but their cellular functions are largely unknown. CoASH can be oxidised to CoASSCoA *in vitro* during prolonged storage in aqueous solutions or in the presence of free radicals. The redox potential of the CoASH/CoASSCoA couple at pH 7.0 is −234 mV and comparable to that of the GSH/GSSG couple (−240 mV), meaning that CoA can significantly contribute to the electrochemical potential inside the cell [34,35]. In bacteria, the CoASH/CoASSCoA ratio is maintained by CoA disulfide reductase which uses NADH (nicotinamide adenine dinucleotide, reduced form) or NADPH to reduce CoASSCoA back to CoASH. Eukaryotic CoA disulfide reductase remains to be identified. The CoA-GSH (CoASSG) mixed disulfide was identified as a renal vasoconstrictor and found to stimulate the proliferation of cultured vascular smooth muscle cells in a dose-dependent manner [36]. Furthermore, CoASSG was shown to activate fructose 1,6-bisphosphatase and to inhibit RNA polymerase [37,38].

The existence of CoA mixed disulfides with cysteine residues in proteins has been known for many years. They were reported in several biochemical and crystallographic studies, and a number of CoA-modified proteins identified as acetyl CoA acetyltransferase, glutamate dehydrogenase flavodoxin, phenol sulfotransferase and peroxide sensor OhrR (organic hydroperoxide resistance repressor) [39–42]. However, the extent of covalent protein modification by CoA, its regulation by oxidative and metabolic stress and the proteome-wide identification of CoA-modified proteins in prokaryotes or eukaryotes have not been investigated until recently.

## Protein CoAlation: a novel post-translational modification associated with redox regulation

Cysteine is one of the most evolutionarily constrained amino acids and the least commonly used in human proteome [43]. Despite this rare usage in protein synthesis, cysteine residues serve critical roles in defining protein structure and function by forming inter- and intramolecular disulfide bonds, coordinating metal ions and participating in catalytic reactions. Furthermore, protein cysteines are targets for numerous post-translational modifications (PTMs) that serve to modulate the activity, regulatory interactions and localisation of diverse proteins. These include S-acylation, oxidation, S-nitrosation, persulfhydration and S-thiolation [44]. The diverse functionality of cysteine residues in proteins is due to the high reactivity of its side chain sulfhydryl group, especially in a biologically oxidative environment. During oxidative stress, the thiol group of cysteine can become progressively oxidised to sulfenic, sulfinic or sulfonic states [44]. The latter modification is irreversible and may lead to the loss of protein function and subsequent degradation. Alternatively, oxidation of cysteine thiols to sulfenic acid acts as a redox switch which facilitates the formation of mixed disulfides with LMW thiols, protecting protein-susceptible thiols from irreversible overoxidation.

In the last decade, proteomics studies have revealed that cysteine modifications involved in protein redox regulation are more widespread in biological systems than previously estimated and exert a considerable influence on cellular processes, including cellular signalling, proliferation, differentiation and apoptosis. A novel mode of redox regulation involving covalent modification of cellular proteins by CoA has recently been discovered in a collaborative effort of several laboratories and termed protein CoAlation [45]. To discover and characterise protein CoAlation *in vitro*, cell-based and animal models, several research tools and methodologies have been developed. These include: (a) anti-CoA monoclonal antibodies, which specifically recognise CoA in various immunological assays, including ELISA, Western blotting, immunoprecipitation and immunohistochemistry; (b) a robust mass spectrometry-based methodology for the identification of CoAlated proteins; and (c) efficient *in vitro* CoAlation assay [45,46]. Analysis of protein CoAlation in a panel of primary and established cell lines treated with oxidising agents revealed that the extent of covalent protein modification by CoA correlates with the level of this coenzyme in cells. This was evidently demonstrated when protein CoAlation was examined in parental and PANK1β overexpressing HEK293 cells (overexpression of PANK1β leads to a ∼6–8 folds increase in the CoASH level). Furthermore, extensive protein CoAlation was observed in rat heart perfused with 100 μM H_2_O_2_. In this experimental model, the patterns of protein CoAlation and protein glutathionylation diverged significantly, suggesting differential targeting of cysteine thiols in cellular proteins by CoA and GSH in response to oxidative stress [45].

Protein CoAlation was also found to be modulated by metabolic stress induced by nutrient deprivation or overload. A significant increase in the level of CoAlated proteins was observed in cells cultured in medium lacking pyruvate and glucose or in rat liver after fasting for 24 h [45]. Feeding rats high-fat/high-sucrose diet for 1 week resulted in a substantial decrease in protein CoAlation in the liver. This finding correlated with a markedly decreased protein CoAlation in the liver of genetically obese ob/ob mice [45].

Extensive protein CoAlation induced by oxidising agents and metabolic stress, and the developed methodology allowed the identification of over 500 CoA-modified proteins in mammalian cells and tissues [45, unpublished observation]. The vast majority of CoAlated proteins were found to be metabolic enzymes (over 65%), as well as proteins implicated in stress response and protein synthesis. Bioinformatics analysis revealed that CoA-modified cysteine residues are frequently found at functionally and structurally important sites in proteins where they participate in a wide variety of biological functions, such as enzymatic catalysis, structure stabilisation, signal transduction, metal binding, PTMs and others.

The p*K*_a_ values of cysteine residues are of critical importance in redox-regulated processes. At physiological pH, the p*K*_a_ values of protein cysteine thiols vary from 8.2 to 9.9 for solvent-accessible cysteines and therefore have low deprotonation ability [47]. Cysteine thiols within a basic three-dimensional environment (low p*K*_a_) are more susceptible to deprotonation and oxidation to sulfenic acid in the presence of ROS [48]. Sulfenic acid is highly reactive and therefore can be further oxidised by ROS to the sulfinic or sulfonic states, if not protected by LMW thiols. Bioinformatics analysis of CoAlated peptides revealed the prevalence of hydrophobic and positively charged amino acids flanking modified cysteines (S. Das et al., unpublished observations). Based on these findings and efficient non-enzymatic *in vitro* CoAlation of metabolic and signalling proteins, we propose that under oxidative stress, CoASH can modify covalently cysteine residues within a basic amino acid environment, as they have lower p*K*_a_ and higher reaction rates with H_2_O_2_ and other oxidising compounds.

A significant difference in the patterns of CoAlated and glutathionylated proteins in H_2_O_2_-perfused rat hearts clearly suggests that different regulatory cysteines can be specifically targeted by CoA and GSH in response to oxidative stress. Bioinformatics of experimentally identified glutathionylation sites revealed the presence of positively charged amino acids (Arg, His or Lys) flanking modified cysteines [49]. Redox-induced covalent attachment of bulky and charged CoA to the thiol group of specific cysteine residues is most likely co-ordinated by neighbouring amino acids in a 3D environment, and the structural correlations and the consensus motif of identified CoAlation sites remain to be determined.

## Functional relevance of protein CoAlation

**Figure 3.**
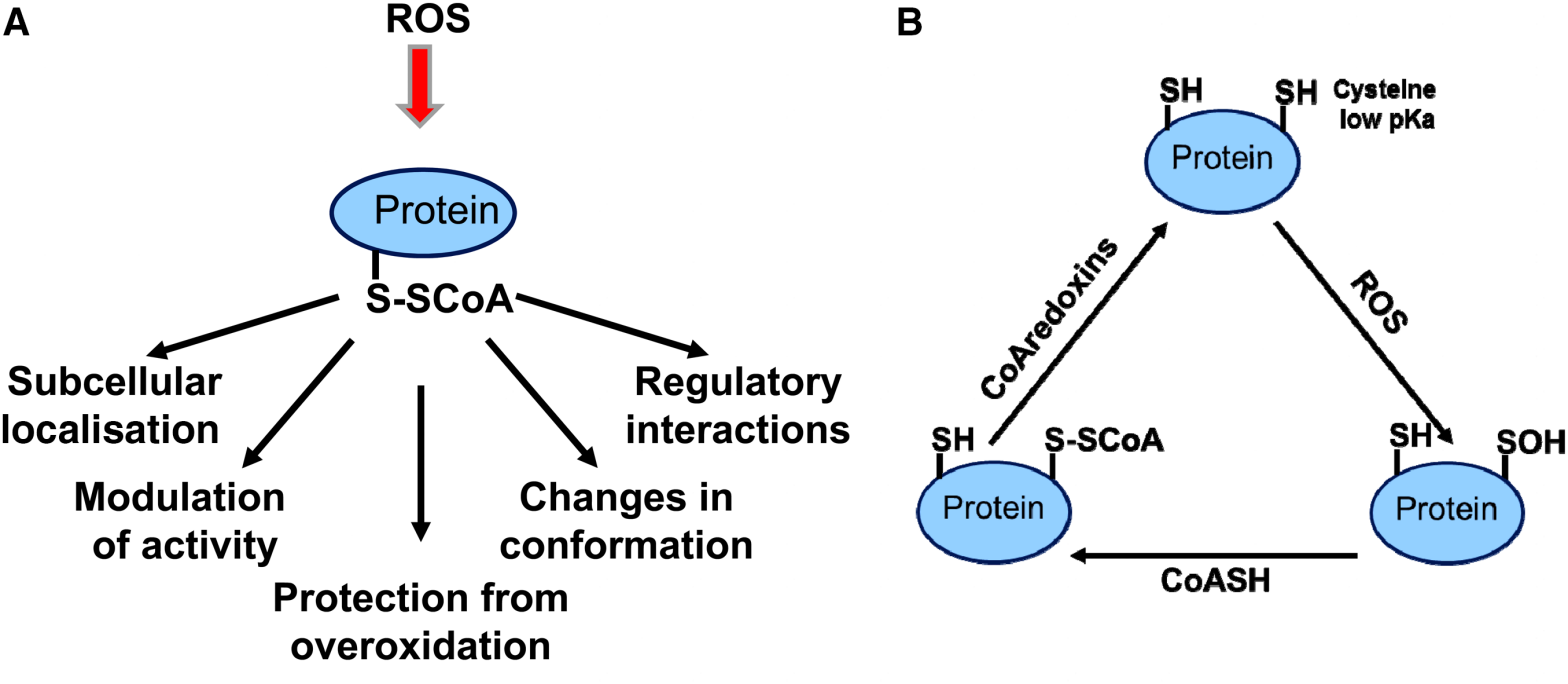
Emerging functions of protein CoAlation in mammalian cells. (**A**) The effect of CoAlation on the function of modified proteins. (**B**) Redox regulation of the protein CoAlation/deCoAlation cycle.

The question which arises from these findings is: how does covalent attachment of CoA affect the regulation and function of modified proteins (Figure 3A)? First of all, enzymatic activities of several metabolic and signalling proteins were found to be negatively or positively modulated by CoAlation. Secondly, the addition of the bulky and charged CoA molecule (767 Da) to thiolate anions of cysteines in target proteins may modulate their stability and subcellular localisation. Thirdly, the presence of pantetheine and 3′5′-ADP moieties on CoA-modified proteins has a potential to generate a unique binding motif for intra- and intermolecular interactions, promoting the formation of regulatory complexes. And finally, the fast and reversible nature of protein CoAlation was observed in mammalian cells exposed to oxidative and metabolic stress [45].

What is the relevance of protein CoAlation in the function of mammalian cells? It is reasonable to speculate that under normal growth conditions, CoA functions to produce metabolically active thioesters, while it may act as a LMW antioxidant protecting protein cysteine thiols from irreversible overoxidation in cellular response to oxidative and metabolic stress.

Maintaining a balance between oxidation and reduction reactions is essential for key cellular processes, such as growth, proliferation, differentiation and survival. Accumulating evidence indicates that dysregulation of redox-sensitive signalling and the thiol–disulfide homeostasis are associated with various human pathologies, including metabolic syndrome, cancer, cardiovascular and neurodegenerative diseases. Protein CoAlation is an emerging field of research that has the potential to become an integral part of redox sensing and regulation under physiological and pathophysiological conditions.

## Conclusions and future perspectives

Understanding how mammalian cells sense ROS and co-ordinate downstream biological responses is still a major challenge. The original findings on protein CoAlation raise a fundamentally important question: what are the molecular mechanisms of protein CoAlation/deCoAlation? The proposed redox-regulated CoAlation/deCoAlation cycle is shown in Figure 3B. Efficient protein CoAlation can be achieved *in vitro* by a non-enzymatic mechanism [45]. However, it is reasonable to speculate that the conjugation of CoA to protein cysteine thiols can be enzymatically enhanced, as reported for GSTs and protein glutathionylation. Preliminary studies in our laboratory indicate that the removal of CoA from covalently modified proteins is enzymatically mediated. We suggest that protein CoAlation is reversed by the action of CoAredoxins (by the analogy to glutaredoxins) and their identities and specificities remain to be determined.

We have recently demonstrated that *in vitro* CoAlation of *S. aureus* glyceraldehyde-3-phosphate dehydrogenase efficiently protects the catalytic cysteine 151 from irreversible overoxidation by H_2_O_2_ (Y. Tsuchiya, unpublished observations). The potential for CoA to function as a physiological antioxidant in cell-based and animal models has yet to be investigated.

Developing new research tools and methods will be instrumental for promoting research on protein CoAlation. The quantitative measurement of protein CoAlation in proteome-wide studies and the *in situ* visualisation of CoAlated proteins in cells and tissues will be of particular importance for advancing research in this emerging field of study.

Unravelling the roles of protein CoAlation and antioxidant function of CoA in human pathologies associated with oxidative stress and redox imbalance is an important goal for the future.

## Abbreviations

4′-PP: 4′-phosphopantetheine
CoA: coenzyme A
COASY/CoAsy: CoA synthase
DPCK: dephospho-CoA kinase
ENPP: Ectonucleotide pyrophosphatase
GSH: glutathione
LMW: low molecular weight
PANK: pantothenate kinase
PPAT: 4′-phosphopantetheine adenylyltransferase
PPCDC: 4′-phosphopantothenoylcysteine decarboxylase
PTMs: post-translational modifications
RES: reactive electrophilic species
RNS: reactive nitrogen species
ROS: reactive oxygen species
